# High Prevalence of Hypovitaminosis D in Institutionalized Elderly Individuals is Associated with Summer in a Region with High Ultraviolet Radiation Levels

**DOI:** 10.3390/nu11071516

**Published:** 2019-07-04

**Authors:** Sara Estéfani S. Sousa, Márcia Cristina Sales, José Rodolfo T. Araújo, Karine C.M. Sena-Evangelista, Kenio C. Lima, Lucia F.C. Pedrosa

**Affiliations:** 1 Postgraduate Program in Nutrition, Federal University of Rio Grande do Norte, Av. Senador Salgado Filho, 3000, Lagoa Nova, 59078970 Natal, RN, Brazil; 2 School of Medicine, State University of Roraima, Rua Sete de Setembro, 231, Canarinho, 69306530 Boa Vista, RR, Brazil; 3 Postgraduate Program of Health Sciences, Federal University of Rio Grande do Norte, Rua General Gustavo Cordeiro de Farias, Petrópolis, 59010180 Natal, RN, Brazil; 4 Department of Nutrition, Federal University of Rio Grande do Norte, Av. Senador Salgado Filho, 3000, Lagoa Nova, 59078970 Natal, Brazil; 5 Department of Dentistry, Federal University of Rio Grande do Norte, Av. Senador Salgado Filho, 1787, Lagoa Nova, 59056000 Natal, RN, Brazil

**Keywords:** vitamin D, elderly individuals, nursing homes

## Abstract

Vitamin D may play a significant role in regulating the rate of aging. The objective of the study was to assess vitamin D status and its associated factors in institutionalized elderly individuals. A total of 153 elderly individuals living in Nursing Homes (NH) were recruited into the study. Serum 25-hydroxyvitamin D [25(OH)D] concentration was used as the biomarker of vitamin D status, and it was considered as the dependent variable in the model. The independent variables were the type of NH, age-adjusted time of institutionalization, age, sex, skin color, body mass index, waist and calf circumference, physical activity practice, mobility, dietary intake of vitamin D and calcium, vitamin D supplementation, use of antiepileptics, and season of the year. Serum 25(OH)D concentrations less than or equal to 29 ng/mL were classified as insufficient vitamin D status. The prevalences of inadequate dietary intake of vitamin D and calcium were 95.4% and 79.7%, respectively. The prevalence of hypovitaminosis D was 71.2%, and the mean serum concentration of 25(OH)D was 23.9 ng/mL (95% confidence interval [CI]: 22.8–26.1). Serum 25(OH)D concentration was associated with the season of summer (*p* = 0.046). There were no associations with other independent variables (all *p* > 0.05). The present results showed that a high prevalence of hypovitaminosis D was significantly associated with summer in institutionalized elderly individuals.

## 1. Introduction

The population aged 60 or over is growing at a faster rate than the total population in almost all regions worldwide. Most notably, the current challenge in demographics is the rapidly increasing population of older adults. In 2012, people aged 60 or over represented almost 11.5 per cent of our total global population of 7 billion. By 2050, the proportion is projected to nearly double to 22 per cent [1].

The number of elderly individuals in Brazil was estimated to be more than 30.2 million in 2017. Thus, they represented approximately 7 per cent of the Brazilian population of 209.3 million inhabitants in this period. In the Northeast of Brazil, the proportion of elderly individuals increased from 5.8% in 2000 to 7.2% in 2010 [2].

A widespread health problem experienced among elderly individuals is multiple micronutrient inadequacy, including vitamin D, which may lead mainly to frailty, sarcopenia, and cognitive decline [3,4].

Vitamin D regulates aging by controlling the activity of autophagy, which slows down aging processes by removing dysfunctional mitochondria. Vitamin D also reduces mitochondrial dysfunction, oxidative stress, inflammation, calcium signaling, epigenetic changes and DNA disorders, including telomere shortening, which act upon aging processes [5].

The diagnosis of vitamin D deficiency is a controversial issue. Serum levels of 25-hydroxyvitamin D [25(OH)D] above 30 ng/mL are associated with increased efficacy of intestinal calcium absorption and stabilization of serum parathyroid hormone (PTH) values, which are suggested as being the threshold for fracture prevention [6]. The Endocrine Society suggests that the 25(OH)D concentrations that are considered deficient, insufficient, and sufficient are ≤20, ≥21 and ≤29, and ≥30 ng/mL, respectively [7]. However, the Brazilian Society of Clinical Pathology/Laboratory Medicine and the Brazilian Society of Endocrinology and Metabolism recently proposed concentrations of 25(OH)D above 20 ng/mL as being adequate for the health of the population up to 60 years old, while values between 30 and 60 ng/mL are adopted for groups at risk of vitamin D deficiency, including elderly individuals [8].

Vitamin D deficiency among elderly individuals can result in cardiovascular risk, mortality, low quality-of-life scores, decreased physical functionality, secondary hyperparathyroidism, and increased risk of fractures [3,9,10,11]. Age-related changes in body composition, such as decreased muscle mass and increased adipose tissue, can lead to a decrease in serum vitamin D concentrations. Furthermore, the muscular weakness presented by the elderly population can be potentiated by the deficiency of vitamin D [12]. Moreover, Pilz et al. (2012) observed a significantly increased mortality risk in the female Nursing Home (NH) residents with the lowest 25(OH)D levels [13].

Several factors are involved in changes in vitamin D status in aging, such as the seasonality, which is currently observed as being an important predictor because it has an impact on the behavior and lifestyle of individuals [6,14,15]. Even under favorable environmental conditions, vitamin D bioavailability in elderly individuals can be negatively affected by the reduced capacity of its cutaneous synthesis. Furthermore, this population can be vulnerable to vitamin D deficiency as a result of low exposure to sunlight, their particular type of skin pigmentation, adiposity, use of antiepileptic medications, reduced kidney function, and low dietary vitamin D intake [9,15,16,17].

In light of these considerations, hypovitaminosis D has been frequently found among institutionalized elderly individuals living in countries with low ultraviolet radiation (UV) indexes, where elderly people spend more time indoors [13,18], as well as in high-latitude regions where there is a high UV index [16,19,20].

Although several studies carried out on elderly people have revealed a poorer vitamin D status during the winter seasons because of the lower sunlight exposure [19,20,21,22], contradictory findings have been found in the literature. One study developed with elderly people from a Mediterranean area suggested significant differences in serum 25(OH)D concentrations between seasons, with the lowest concentrations occurring in the summer and the highest during the autumn, although their vitamin D intake was significantly lower in the autumn and winter [23].

Based on these findings, our study was developed to answer the question about the status of vitamin D in institutionalized elderly individuals living in a region with high levels of UV radiation, while considering various particularities of aging. For this reason, we have drawn up a theoretical model that encompasses diverse variables and contemplates these conditions.

In addition to this, institutionalization impacts the interactions of elderly individuals with the environment as well as their nutritional status, which, in turn, are influenced by their social status, psychological disorders, and general health conditions [24,25]. In this scenario, the objective of the present study is to assess serum 25(OH)D concentrations and associated factors in institutionalized elderly individuals living in a region of northeastern Brazil with high ultraviolet radiation levels.

## 2. Materials and Methods

### 2.1. Study Population

**Figure 1:**
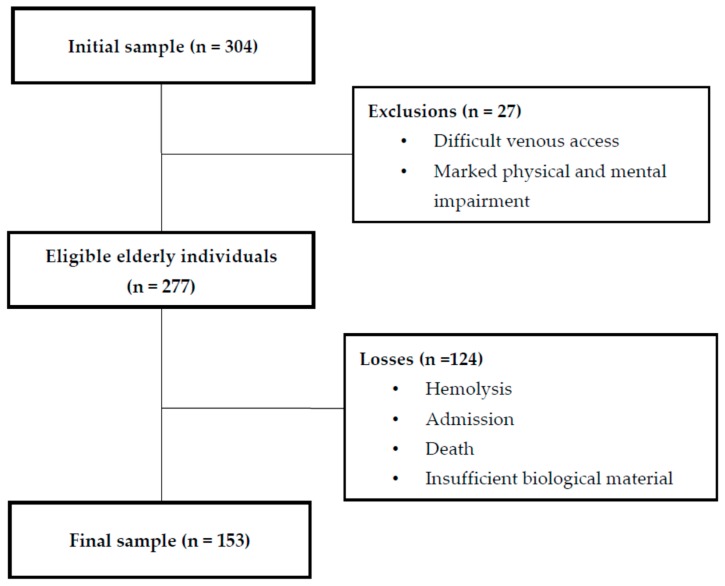
Flowchart of the study participants.

The study was carried out with 304 elderly individuals living in nine Nursing Homes (NH) in the city of Natal (Rio Grande do Norte state), which is in the northeast of Brazil. All the individuals included in the study were at least 60 years old and living in these NHs. In Brazil, the Statute of the Elderly [26] considers elderly individuals to be aged 60 years or older. Elderly individuals who had marked physical and mental impairments, as well as difficult venous access, were excluded from the study. After exclusion, the initial sample of 304 elderly individuals was narrowed down to 153 participants (Figure 1).

The study was conducted in accordance with the Declaration of Helsinki, and the protocol was approved by the Research Ethics Committee of the Federal University of Rio Grande do Norte (#263/11; CAAE 0290.0.051.000-11). All participants or their guardians provided written informed consent before they participated in the study.

The sample size calculation for the cross-sectional studies was defined on the basis of the mean and standard deviation of the concentration of 24.0 (7.6) ng/mL of 25(OH)D, corresponding to 10 random subjects in the sample of this study, and to the power of the test of 95%, resulting in 154 elderly individuals. Thus, this study obtained a response rate of 99.3%. For association studies, the sample size would be 156 elderly individuals, considering the population size of 304 elderly individuals, a hypothetical frequency of vitamin D deficiency of 71.2% [19], a confidence limit of 5%, and a design effect equal to 1 [27].

### 2.2. Theoretical Model of the Study

**Figure 2:**
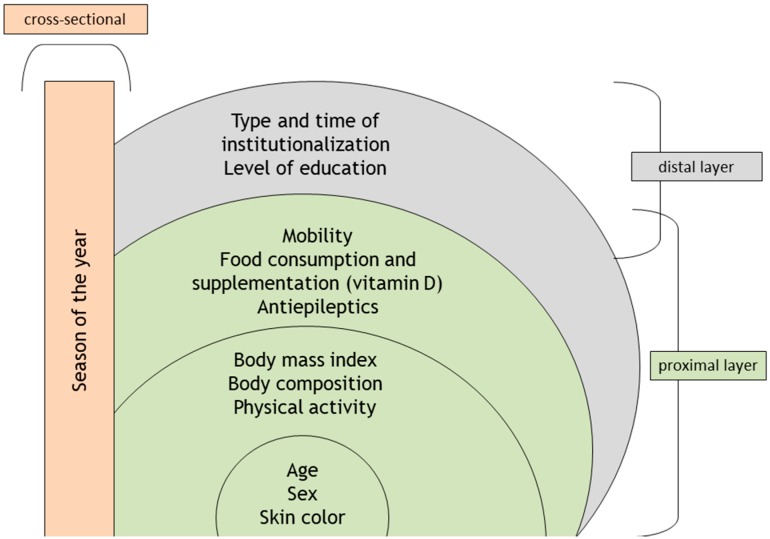
Theoretical model of the determinants of vitamin D status in institutionalized elderly individuals.

The theoretical model of this study was developed according to determinant factors of vitamin D status. Serum 25(OH)D concentration was defined as the dependent variable, and the independent variables were those likely to influence vitamin D status: Age, sex, skin color, body mass index (BMI), physical activity practice, mobility, diet, use of vitamin D supplements, and use of antiepileptic medications. The sociodemographic, nutritional, lifestyle, and biological variables were included in the “proximal” layers. Type of NH, age-adjusted time of institutionalization, and level of education were components of the “distal” layer. The season of the year was considered as a cross-sectional influence because it has a direct relationship with 25(OH)D concentrations (Figure 2). This study also addressed the relationship between the dependent variable and possible clinical implications in cases of vitamin D-deficient elderly individuals—multimorbidity, falls, sarcopenia, depression, cognition, and functional status.

Age-adjusted time of institutionalization was calculated to determine how much time after the advanced age period (as a percentage, defined as the time after turning 60 years of age) elderly individuals remained institutionalized, according to a previous study [28].

Weight, height, and waist and calf circumference were measured to determine the anthropometric variables. Skin color was obtained by self-classification among the five categories adopted by the Brazilian Institute of Geography and Statistics (IBGE): Black, mixed race (“pardo” in official Portuguese), Caucasian, Asian, and indigenous (Native Brazilian) [29]. Body weight was assessed with a Balmak^®^ electronic scale (Santa Barbara do Oeste, Brazil) with a capacity of 300 kg and an accuracy of 50 g. In elderly individuals with disabilities or permanent mobility restrictions, weight was measured with a Seca^®^ 985 bed scale (Bolton, England). Height was defined using the average of the two measurements and a portable Caumaq^®^ stadiometer (Cachoeira do Sul, Brazil). For physically impaired elderly individuals, height was estimated using the equation of Chumlea et al. (1987) [30]. BMI was classified while considering the cutoff points proposed by Lipschitz [31].

Calf perimeter values less than 31 cm were indicative of loss of muscle mass in elderly individuals. Results for waist perimeter greater than 102 cm in males and 88 cm in females were considered to indicate a higher risk of diseases [32].

Physical activity practice was determined through information provided by caregivers, categorized into “yes” or “no” answers. Information on diseases, multimorbidity, polypharmacy, use of antiepileptic drugs, and vitamin D supplementation was collected from the medical records of the NHs. Multimorbidity was considered as the presence of two or more diseases with clinical diagnoses included in medical records. Polypharmacy was characterized for those individuals who took five or more medications daily [33].

Data on the mobility variable were collected from the caregivers of the elderly individuals using a Barthel scale [34]. Sarcopenia was measured according to previously defined criteria [35], based on discrimination of reduced muscle mass concentrations (calf circumference assessment) associated with a reduction in strength (grip test) and/or functional status. Functional status was assessed according to the scale set by Katz [36]. Cognitive function was assessed according to Pfeiffer’s Short Portable Mental Status Questionnaire, which evaluates short- and long-term memory, orientation, information on everyday facts, and mathematical reasoning. Individuals were classified into four categories: intact mental function (0–2 errors), mild cognitive impairment (3–4 errors), moderate (5–7 errors), and severe (8–10 errors) [37,38].

The elderly subjects were categorized by seasons. The season used was the date corresponding to 30 days prior to blood collection. This was to take into account the half-life of 25(OH)D [39]. The Laboratory of Environmental and Tropical Variables of the Federal University of Rio Grande do Norte provided UV values for each season of the year [39].

### 2.3. Analysis of 25(OH)D Serum Concentration

Blood samples after overnight fasting (8–12 h) were collected by standard venipuncture. Serum concentrations of 25(OH)D were measured using the chemiluminescent Liaison^®^ test from kit DiaSorin^®^ (Saluggia, Italy). Serum 25(OH)D values equal to or below 29 ng/mL (72 nmol/L) were considered as “insufficient”, while values between 30–60 ng/mL (75–150 nmol/L) were classified as “sufficient” [8]. In the present study, the name “hypovitaminosis D” was used for elderly individuals with a 25(OH)D value equal to or below 29 ng/mL.

### 2.4. Dietary Intake

Food consumption data were collected according to the method of weighed food records (WFRs), as described by Sales et al. (2018) [28]. Habitual intake of each nutrient was estimated by removing the effect of intrapersonal variance, based on the method developed by Nusser et al. (1996) [40]. Subsequently, the results were adjusted for energy intake [41]. The prevalences of inadequate intake of calcium and vitamin D were estimated by using the Estimated Average Requirement (EAR) as the cut-off point [42].

### 2.5. Statistical Analysis

The data were analyzed using the statistical software IBM SPSS Statistics 21 (IBM Corp, Armonk, NY, USA). Initially, a descriptive analysis was performed with variables at the individual level, which allowed the sample to be characterized for socioeconomic, health, anthropometric, nutritional, and vitamin D statuses. Data on serum levels of 25(OH)D and dietary intake were checked for missing data and outliers that could hinder a multivariate analysis. In addition, variables were excluded from further analysis when they had more than 25% of missing data and outliers, identified under a multivariate perspective through measurement of the Mahalanobis distance (D^2^)—distance greater than 3.0. There was no record of sample losses and multivariate outliers after adjustment of intrapersonal variability, interpersonal variability, and energy from dietary intake. The normality was checked by using the Kolmogorov–Smirnov test. Subsequently, the variables color, body mass index, cognition, functional status, and seasons of the year were dichotomized according to the predominance of elderly individuals in groups and/or clinical characteristics. Subsequently, to check for statistical associations, Pearson’s chi-square test was run, considering the outcome variable (25(OH)D) and other variables relative to the theoretical model. The magnitude of the association was assessed using prevalence ratios (PR) with the corresponding confidence intervals (CIs). The significance level α was set at *p* < 0.05 (two-tailed test).

## 3. Results

The power of the study was 99.7% (with continuity correction), considering a 95% confidence interval. The calculation was obtained from 109 exposed participants and 44 unexposed participants, resulting in a prevalence of vitamin D insufficiency of 71% and 28%, respectively. Thus, the prevalence ratio (PR) was equal to 2.5, with a difference of 43% among the prevalences.

The mean age of the elderly individuals was 81.7 (9.2) years, ranging from 60–103. Thus, they were a long-living population, and there were more females (78.4%) than males. It was found that 64.5% of them had some level of education, 70.6% lived in nonprofit institutions, and that the average age-adjusted time of institutionalization was 29.4%. The BMI values indicated that 46.4% of the elderly individuals were thin.

It was found that 60.3% showed signs of loss of muscle mass, according to calf circumference. Most of the elderly individuals did not practice physical activity, had moderate-to-severe cognitive impairment, and showed multimorbidity (Table 1).

Concerning functional status, 31.8% of the elderly individuals were classified as “A” (independent for eating, continence, locomotion, toilet use, dressing, and bathing), followed by 16.2% in the item “G” (dependent on performing the above six functions). Dietary intake of vitamin D was 2.8 (3.2) µg/day, resulting in a high prevalence of inadequacy (95.4%), in addition to low frequency in the use of vitamin D supplementation (5.9%). The prevalence of hypovitaminosis D in the study population was 71.2%, with a mean value of 23.9 (16.6) ng/mL for 25(OH)D/mL. No participant was categorized in the winter season according to the criteria applied to the distribution of elderly individuals by seasons (Table 2).

There was an association between 25(OH)D and summer (*p* = 0.046), and there were no associations with any other independent variables (all *p* > 0.05) (Table 3).

## 4. Discussion

The high prevalence of hypovitaminosis D found in the institutionalized elderly individuals of the present study was only significantly associated with the summer season, and this was found in the theoretical model involving several proximal and distal factors that determine vitamin D status. Moreover, the seasons of the year were considered as cross-sectional variables. The evaluation of the seasons of the year together with the assessment of the dietary intake of vitamin D (whether it was adequate or inadequate) was a relevant point of the present study, in that it was necessary to consider the two external sources of vitamin D obtained by the population.

Vitamin D is the only nutrient acquired from external sources, e.g., natural sunlight. It is estimated that an individual’s highest daily vitamin D intake can be obtained by exposure to sunlight. However, in elderly individuals, the capacity of the cutaneous synthesis of vitamin D is undermined by the gradual loss of this physiological mechanism [9,17].

In epidemiological studies on vitamin D status, physical activity is often used as a proxy for time spent outdoors and, indirectly, for exposure to sunlight. Consequently, the elderly individuals who spend their lives at home indoors and those who spend less time doing outdoor physical activities have decreased levels of 25(OH)D [43].

Many of the elderly individuals were distributed by the summer season (60.1%), but although the region where the study was conducted has an annual average of UV indexes ranging from high to very high [44], it can be inferred that the studied population did not benefit from this environmental factor, as shown by the contradictory significant association between hypovitaminosis D and summer. We know that in tropical regions, the summer heat is extremely uncomfortable, causing most elderly individuals to avoid sunlight exposure. Similar reasons for this paradoxical finding were previously discussed, emphasizing different weather conditions between areas or countries [23]. In addition, their failure to benefit from the summer season can also be explained because activities in the NHs follow a pattern―they start early in the day with personal hygiene and provision of food and medicines, and end with little or no sunlight exposure, because the times are inappropriate. Furthermore, factors such as health problems, logistics of the NH, and indoor leisure activities affect the access of the elderly individuals to sunlight exposure more often when compared to their non-institutionalized peers [19,45].

Other findings from our study, such as the high inadequacies of dietary vitamin D and calcium, physical inactivity, and multimorbidities may additionally have nullified the response to environmental conditions that would otherwise be favorable for vitamin D status. In regard to elderly Brazilian subjects, even in the summer, Caucasian males in the youngest group of individuals presented a significant increase in 25(OH)D levels, demonstrating that a seasonal variation in 25(OH)D concentrations was influenced by factors such as gender, ethnicity, and age [20].

Okan et al. noted that 25(OH)D levels and the UV index of elderly people living in NHs were significantly lower than those living in their own homes, though there was a positive correlation between UV index and 25(OH)D observed in both groups [46].

These data reinforce the need to discuss vitamin D status in the context of the institutionalization of elderly individuals, and demands a model that encompasses diversified variables, such as those used in our study.

Although aging has been reported as a factor that interferes with vitamin D metabolism [9,17], the 25(OH)D concentrations in elderly Brazilian individuals with regular sunlight exposure and physical activity practice were similar to the values found in young and healthy individuals. These results confirm that vitamin D metabolism is activated by sunlight exposure, despite the physiological limitations of elderly individuals [20]. Physical activity is considered to be one of the factors associated with higher 25(OH)D levels [14]. In light of this evidence, some benefits can be achieved with changes in the lifestyles of elderly individuals.

Most of the elderly individuals lived in nonprofit institutions and had lived 29% of their life as an elderly individual in an institutional setting, which was considered a distal factor of the study model and was associated with an increased risk of vitamin D insufficiency and hypovitaminosis D when compared to elderly individuals living in the community [19]. In the same study, the prevalences of hypovitaminosis D in institutionalized elderly individuals and outpatients from a city in southeastern Brazil were 71.2% and 43.8%, respectively. This result is similar to that found in the present study. In this regard, confounding factors arising from disease burden in elderly individuals may also account for the high prevalence of vitamin D insufficiency. In a previous study, frail elderly individuals who had been recently institutionalized showed a 41% prevalence of vitamin D deficiency and a 45% prevalence of vitamin D insufficiency [47].

In our study, the population profiles and the infrastructures of the NHs were similar. Therefore, the studied elderly individuals did not show significant differences, whether they were in nonprofit public or for-profit private institutions. As shown in Table 3, the high prevalence of hypovitaminosis D in for-profit private institutions demonstrates that the institutionalization, by itself, can be addressed as a determinant of health problems in NHs. Furthermore, the study population lives in the Northeast of Brazil, a region with high social inequalities that may affect genetic distinctions in comparison to other areas of the country. A study developed in the same region found evidence that longevity in this region was characterized by the presence of longer telomeres in women associated with a low level of education [48].

All these factors may partly explain the findings reported in this study. However, the high prevalence of vitamin D insufficiency, per se, in the context of low dietary intake of vitamin D and calcium as well as physical inactivity, represents signs of deterioration in vitamin D status, thus affecting the health of institutionalized elderly individuals.

Other points should be addressed in the field of aging. This study focuses on this particular generation of long-living individuals, and according to the theoretical model proposed, age is a proximal factor of interference in 25(OH)D concentrations. In this regard, there is increasing evidence that aging can proceed at variable rates and be regulated by vitamin D. Normal concentrations of vitamin D are capable of maintaining these processes at their regular low rates, and this slows down the aging process and also helps to prevent the onset of some age-related diseases. When vitamin D is deficient, there is an increase in the activities of these aging processes, which not only accelerates the rate of aging, but also creates conditions that initiate the onset of age-related diseases [5].

The present study showed limitations regarding its temporal approach (cross-sectional), hindering the establishment of a causal relationship, and also due to lack of evaluation of sun exposure. Future studies should focus on reproducibility, involving a protocol with longitudinal observation and comparison among institutionalized and non-institutionalized elderly individuals. Furthermore, as shown by the benefits found in institutionalized elderly individuals in Finland [49] who received vitamin D food fortification, the literature reinforces the need for special attention in health care for elderly people, with a focus on modifiable risk factors.

## 5. Conclusions

A high prevalence of hypovitaminosis D was found in institutionalized elderly individuals living in a region with high ultraviolet radiation levels, and this was significantly associated with summer. These findings suggest the probability of the presence of interfering vitamin D factors mediated by aging. In this scenario, aging conditions which increase the risk of vitamin D deficiency have to be taken into consideration. Furthermore, the institutionalization of elderly individuals needs to be carefully handled to create alternatives that mitigate aging-related health problems.

## Figures and Tables

**Table 1:** General, anthropometric, lifestyle, and health characteristics of institutionalized elderly individuals (*n* = 153).

Variables	*n* (%)	95% CI
Sex		
Male	33 (21.6)	3.30–15.00
Female	120 (78.4)	3.30–71.90
Skin color ^a^		
White	70 (54.7)	46.10–54.10
Black	25 (19.5)	12.50–26.60
Brown	27 (21.1)	14.10–28.90
Other	6 (4.7)	1.60–8.60
Marital status ^b^		
Single	66 (52.0)	42.50–60.60
Widowed	32 (25.2)	18.10–33.10
Married	17 (13.4)	7.90–19.70
Separated/Divorced	12 (9.4)	3.70–17.30
Level of education ^c^		
Illiterate	43 (35.5)	27.30–43.80
Literate	32 (26.4)	18.20–34.70
Elementary I (years 1–5)	16 (13.2)	6.60–19.00
Elementary II (years 6–9)	6 (5.0)	1.70–9.10
High School	18 (14.9)	8.30–21.50
Higher Education	6 (5.0)	1.70–9.10
Type of institution		
Profit	45 (29.4)	22.20–37.20
Nonprofit	108 (70.6)	62.80–77.80
Time of institutionalization (mean) ^d^	29.38%	24.72–34.04
BMI (kg/m²) ^e^		
Thin	55 (46.4)	39.10–54.00
Normal weight	37 (31.1)	23.90–39.50
Excess weight	27 (22.7)	15.10–29.40
Abdominal circumference ^f^		
Obese	51 (40.5)	31.80–50.00
Non-obese	75 (59.5)	50.00–68.20
Calf circumference ^g^		
Sign of lean mass loss	88 (60.3)	51.10–68.50
No sign of lean mass loss	58 (39.7)	31.50–47.90
Physical activity ^h^		
Inactive	87 (70.2)	67.80–81.50
Active	37 (29.8)	18.50–32.20
Mobility		
Without mobility	20 (13.1)	7.80–19.00
With mobility	133 (86.9)	81.00–92.20
Sarcopenia ^g^	66 (45.2)	46.60–63.00
Cognition ^i^		
Preserved mental function	13 (9.0)	4.90–13.90
Mild cognitive impairment	9 (6.3)	2.80–10.40
Moderate cognitive impairment	26 (18.1)	11.80–25.00
Severe cognitive impairment	96 (66.7)	58.40–74.30
Multimorbidity ^b^	84 (66.1)	57.50–74.00
Polypharmacy ^j^	59 (38.6)	34.80–50.70
Use of antiepileptic medications	27 (18.8)	12.50–25.70

^a^ n = 128; ^b^ n = 127; ^c^ n = 121; ^d^ n = 133; ^e^ n = 151; ^f^ n = 126; ^g^ n = 146; ^h^ n = 124; ^i^ n = 144; ^j^ n = 139.

**Table 2:** Dietary and environmental characteristics of institutionalized elderly individuals (*n* = 153).

Variables	Mean (SD)	95% CI
Vitamin D intake (µg/day)	2.8 (3.2)	2.32–3.33
Calcium intake (mg/day)	997.9 (291.8)	921.18–1074.64
Serum 25(OH)D (ng/mL) ^a^	23.9 (16.6–31.0)	-
Local UV Index		
Summer	6.6 (0.5)	6.45–6.66
Spring	6.6 (0.5)	6.51–6.80
Fall	6.3 (0.4)	6.48–6.99
Vitamin D intake		
Possibly inadequate	146 (95.4)	92.20–98.70
Possibly adequate	7 (4.6)	1.30–7.80
Calcium intake		
Possibly inadequate	122 (79.7)	73.20–85.60
Possibly adequate	31 (20.3)	14.40–26.80
Vitamin D status ^b^		
Insufficient	109 (71.2)	64.70–78.40
Sufficient	44 (28.8)	21.60–35.30
Distribution by seasons		
Summer	92 (60.1)	51.60–68.00
Spring	46 (30.1)	22.90–37.90
Fall	15 (9.8)	5.20–15.00

^a^ median (inter-quartile range between 25 and 75, respectively); ^b^ according to the diagnosis of vitamin D status.

**Table 3:** Association between serum 25(OH)D concentration and variables of the theoretical model.

	Serum 25(OH)D (ng/mL)					
	Hypovitaminosis D (≤29)		Sufficient (30–60)			
	*n*	%	*n*	%	PR ^a^ (95% CI)	*p*
Sex						
Male	25	75.8	8	24.2	1.08 (0.86–1.36)	0.518
Female	84	70.0	36	30.0		
Age						
75 years or older	84	73.0	31	27.0	1.11 (0.86–1.43)	0.392
60–74 years	25	65.8	13	34.2		
Skin color						
Non-white	36	62.1	22	37.9	0.80 (0.63–1.02)	0.063
White	54	77.1	16	22.9		
Type of institution						
Nonprofit	72	66.7	36	33.3	0.43 (0.18–1.03)	0.053
Profit	37	82.2	8	17.8		
Body Mass Index						
Non-normal weight	83	72.3	31	27.2	0.63 (0.25–1.57)	0.314
Normal weight	30	81.1	7	18.9		
Abdominal circumference						
With abdominal fat	39	76.5	12	23.5	0.85 (0.68–1.06)	0.181
Without abdominal fat	49	65.3	26	34.7		
Calf circumference						
Sign of muscle mass loss	62	70.5	26	29.5	1.05 (0.86–1.29)	0.628
No sign of muscle mass loss	43	74.1	15	25.9		
Physical activity						
Inactive	75	68.8	34	31.2	0.43 (0.16–1.12)	0.078
Active	31	83.8	6	16.2		
Mobility						
Yes	17	85.0	3	15.0	1.23 (0.99–1.53)	0.145
No	92	69.2	41	30.8		
Multimorbidity						
Yes	59	70.2	25	29.8	0.91 (0.77–1.23)	0.828
No	31	72.1	12	27.9		
Falls						
Yes	4	80.0	1	20.0	1.12 (0.72–1.76)	0.669
None	99	71.2	40	28.8		
Sarcopenia						
Yes	57	71.3	23	28.8	1.00 (0.81–1.23)	0.996
No	47	71.2	19	28.8		
Cognition						
Moderate to severe impairment	89	73.0	33	27.0	1.33 (0.90–1.99)	0.083
Preserved to mild impairment	12	54.5	10	45.5		
Depression						
Depressed	6	54.5	5	45.5	0.75 (0.13–1.30)	0.198
Non-depressed	86	72.9	32	27.1		
Functional status						
Reduced independence	68	67.3	33	32.7	0.88 (0.71–1.08)	0.251
Independent	36	76.6	11	23.4		
Seasons of the year						
Other seasons	38	62.3	23	37.7	0.49 (0.24–1.00)	0.046
Summer	71	77.2	21	22.8		
Vitamin D intake						
Possibly inadequate	104	71.2	42	28.8	1.00 (0.62–1.61)	0.991
Possibly adequate	5	71.4	2	28.6		
Dietary Calcium						
Possibly inadequate	117	95.9	5	4.1	1.02 (0.93–1.13)	0.576
Possibly adequate	29	93.5	2	6.5		
Vitamin D supplementation						
Yes	87	70.2	37	29.8	1.05 (0.65–1.69)	0.825
No	6	66.7	3	33.3		
Antiepileptic medications						
Yes	19	70.4	8	29.6	0.98 (0.75–1.28)	0.882
No	84	71.8	33	28.2		

^a^ Prevalence Ratio.
